# Relay maize after tobacco enhances rapeseed growth and nutrition by reshaping soil microbial communities in an annual triple cropping system

**DOI:** 10.3389/fpls.2026.1827060

**Published:** 2026-07-02

**Authors:** Yuxin Yang, Yongcong Xia, Yuanyuan Li, Wenwu Li, Jiajie Luo, Cunwu Guo, He Zhang, Bingbing Jiang, Shusheng Zhu, Junying Li, Yixiang Liu, Cuiying Wang

**Affiliations:** 1 Baoshan University, College of Resources and Environment, Baoshan, China; 2 Yunnan Agricultural University, Key Laboratory of Agricultural Biodiversity and Pest Control of Ministry of Education/State Key Laboratory of Conservation and Utilization of Yunnan Biological Resources, Kunming, China; 3 Yunnan Academy of Tobacco Agricultural Sciences, Kunming, China; 4 International Associated Laboratory of China and French in Agriculture, Yunnan Agricultural University, Kunming, China; 5 Linxiang District Agricultural Technology Extension Station, Lincang, China

**Keywords:** growth dynamics, plant nutrition, rapeseed, soil microbial, theoretical yield, triple-cropping system

## Abstract

Multiple cropping systems are increasingly being adopted worldwide to improve land-use efficiency and promote sustainable agriculture. To optimize the traditional post-tobacco fallow period, we evaluated a novel tobacco–maize–rapeseed (T_M_R) triple-cropping system. Using field experiments and high-throughput amplicon sequencing, we investigated the effects of the preceding tobacco crop and the additional maize season on rapeseed performance and soil microbial ecology. The addition of a maize season enhanced early growth vigor of rapeseed and improved key yield components, resulting in a theoretical yield of 5388.48 kg/ha. Rapeseed nitrogen uptake significantly increased compared with the conventional rotation (*P* < 0.05), and was positively associated with soil alkali-hydrolyzable nitrogen and major yield components. Different cropping systems significantly reshaped the β-diversity of soil microbial communities in the root-zone soil of rapeseed. Under the maize addition treatment, bacterial co-occurrence networks exhibited greater connectivity and complexity. *Chujaibacter*, *Sporosarcina*, and *Epicoccum* were enriched and identified as discriminative taxa using random forest analysis. In conclusion, the tobacco–maize–rapeseed triple-cropping system enhances nitrogen nutrition and reshapes soil microbial communities, providing a sustainable strategy for improving crop performance and agroecosystem functioning.

## 1 Introduction

In the face of escalating global food security challenges and the continuous decline in available arable land, optimizing agricultural production systems and enhancing land-use efficiency have become key priorities in modern agricultural development (Savary et al., 2012). However, traditional farming practices characterized by continuous cropping and monoculture are associated with numerous challenges, including inefficient use of cultivated land, excessive consumption of water and fertilizer, soil fertility degradation, increased incidence of pests and diseases, and limited gains in production efficiency (Deng et al., 2017; Gałązka et al., 2017; Li et al., 2022). These constraints have substantially limited the sustainable development of agricultural systems. Consequently, innovative cropping systems capable of intensifying agricultural production while maintaining ecological sustainability are urgently needed. In this context, annual multiple cropping systems have attracted considerable attention as an effective strategy for improving land use efficiency and enhancing system productivity (Li et al., 2014).

Diversified cropping systems, implemented through multiple cropping and rotation, are widely recognized for improving land productivity, optimizing cropping structures, and enhancing soil ecosystem sustainability (Li et al., 2020; Xu et al., 2020; Meng et al., 2023; Ruan et al., 2024). Previous studies have demonstrated that crop rotation systems, including cotton–grain–rapeseed and rapeseed–wheat/maize/rice rotations, can improve land-use efficiency, enhance soil quality, and facilitate nutrient cycling, ultimately increasing crop productivity (Gill, 2018; Zhang, 2020; Hirzel et al., 2023; Town et al., 2023; Dong et al., 2024). Moreover, incorporating rapeseed into rotation systems has been shown to reshape soil microbial community composition, stimulate beneficial microbial functions, and suppress soil-borne pathogens (Fang et al., 2016; Liu et al., 2017; Zhong et al., 2022; Liu et al., 2025a). In Yunnan Province, China, a tobacco–maize–rapeseed (T_M_R) triple-cropping system has been developed to maximize the utilization of the post-tobacco fallow period and residual soil resources. By inserting a short-season maize crop into the traditional tobacco–rapeseed rotation, this system achieves three consecutive crop cycles annually (Wang and Dan, 2015). The tobacco–maize–rapeseed triple-cropping system not only improves land-use efficiency but also enhances soil health and agroecosystem resilience, thereby offering a sustainable model for regional agricultural development (Li et al., 2014). While the agronomic and ecological benefits of diversified cropping systems are well recognized, it remains unclear how the introduction of a maize season within tobacco–rapeseed rotations influences subsequent crop performance and soil microbial communities.

Soil microbial communities are increasingly recognized as important mediators of crop legacy effects in diversified cropping systems (Jiang et al., 2016; Li et al., 2025a; Wang et al., 2026). Soil microorganisms interact with plant roots to regulate nutrient cycling, suppress soil-borne diseases, and enhance plant growth, adaptation, and stress resilience (Ling et al., 2022; Liu et al., 2023). Different rotation and intercropping patterns can reshape microbial community composition and ecological interactions, thereby influencing nutrient availability, crop performance, and soil functioning. For instance, maize-wheat rotation has been shown to significantly improve soil physicochemical properties, enhance nutrient cycling, and increase yields of subsequent crops (Yang et al., 2025; Wei et al., 2026). Additionally, rotation systems involving rapeseed with wheat or maize have been found to enhance microbial diversity, suppress pathogenic microbes, and promote the growth of beneficial microorganisms (Tian et al., 2025). However, it remains unclear whether an additional maize season can reshape soil microbial communities and their links with soil nutrient availability and subsequent rapeseed performance.

Although previous studies have highlighted the agronomic advantages of triple-cropping systems, the underlying links between crop sequence configuration and subsequent crop performance remain poorly understood. To address this knowledge gap, this study investigates the effects of distinct preceding crop sequences involving tobacco and maize on the growth performance, nutrient uptake, yield formation, and soil microbial communities of rapeseed. We hypothesized that: (i) the tobacco–maize–rapeseed triple-cropping system would enhance soil nutrient availability, thereby promoting rapeseed growth and yield formation; and (ii) the triple-cropping system would reshape soil microbial community composition and co-occurrence network structure, leading to the enrichment of specific microbial taxa associated with rapeseed productivity. The findings are expected to provide both a theoretical foundation and practical guidance for the scientific optimization and regional application of this multiple cropping system.

## 2 Materials and methods

### 2.1 Experimental design

The field trial was conducted in Boshang Town, Linxiang District, Lincang City, China, from 2023to 2024 (23°53′N, 100°05′E). The tobacco variety used in this experiment was Yunyan87, while the maize and rapeseed varieties were Wugu 1790 and Deyouzao No. 1, respectively. Plots with similar soil type, fertility, drainage, and other physicochemical characteristics were selected. The initial soil physicochemical properties of the experimental field are provided in **Supplementary Table 1**. The experimental treatments included relay maize cropping after tobacco followed by rapeseed rotation (tobacco-maize-rapeseed, T_M_R), tobacco–fallow–rapeseed rotation (tobacco-fallow-rapeseed, T_F_R), and fallow-maize-rapeseed (F_M_R), in which maize was grown without a preceding tobacco crop. The experiment was arranged in a randomized complete block design (RCBD) with three replicates. Each plot measured 21.6 m², and treatments were randomly assigned within each block. The planting time, growth season and harvest time of all crops were kept consistent across treatments, whereas unplanted plots were maintained as fallow. The cropping schedule was as follows: tobacco was transplanted in mid-April, maize was direct-seeded in early August, tobacco leaves were harvested and cured in early September, and rapeseed was direct-seeded in early December. Tobacco was transplanted at a spacing of 1.2 m between rows and 0.5 m between plants within rows. Maize was interplanted between adjacent tobacco ridges. All treatments received identical field management, including irrigation and pest, weed, and disease control. After harvest, aboveground residues of tobacco and maize were removed from the field, whereas root systems were retained in the soil.

During the tobacco-growing season, commercial organic fertilizer was applied as a basal fertilizer at 1500 kg/ha. Tobacco compound fertilizer was applied at a total rate of 960 kg/ha, including a basal application of 60 kg/ha and four subsequent topdressings. Potassium sulfate was applied twice during the growing season at a total rate of 450 kg/ha. Following tobacco harvest, maize in the T_M_R and F_M_R treatments received three fertilizer applications, including one application of urea (150 kg/ha) at the seedling stage and two applications of maize-specific compound fertilizer and urea (225 kg/ha each) during the vegetative and reproductive growth stages. During the fallow periods of the T_F_R and F_M_R treatments, plots were maintained without crop cultivation and received no fertilizer inputs. All fertilizer applications followed local agronomic recommendations and were standardized among treatments receiving the same crop.

### 2.2 Field investigation

To investigate the effects of the three cropping patterns on rapeseed growth, growth dynamics were monitored under the T_M_R, T_F_R, and F_M_R cropping systems. Field surveys were conducted at approximately 15-day intervals throughout the rapeseed growing season. Based on crop phenological stages, detailed measurements were conducted at 45, 85, 100, 115, 130, and 145 days after sowing. At each sampling time, five representative rapeseed plants were randomly selected from each plot. Plant height was measured in the field, and the plants were subsequently harvested to determine aboveground dry weight after oven-drying to constant weight. Mean values for each plot were used to construct logistic growth curves describing rapeseed growth dynamics (Trinder et al., 2012). Logistic growth models were fitted separately for plant height and aboveground biomass.

### 2.3 Soil sampling

At rapeseed maturity, five soil sampling points were randomly selected within each plot. Root-zone soil was collected by carefully excavating rapeseed plants and gently removing loosely attached bulk soil, retaining the soil closely surrounding the root system. In the multiple cropping system, root-zone soil was specifically collected for analysis, given its potential role in mediating the legacy effects of plant–plant interactions. All samples were immediately transported to the laboratory on ice and stored at -80 °C prior to microbiome analyses.

### 2.4 Determination of rapeseed yield components and theoretical yield

In each experimental plot, a measurement area of 1.44 m² was randomly selected. At rapeseed maturity, five representative plants were selected from each area to assess yield components, including effective plants density, number of pods per plant, number of seeds per pod (calculated as the average of 10 randomly selected pods per plant), and thousand-seed weight (Li et al., 2019). Theoretical yield was calculated as follows: Theoretical yield (kg/ha) = Number of effective plants per hectare × number of pods per plant × number of seeds per pod × 1000-seed weight × 10^-6^ × 0.85, where 0.85 is an empirical correction coefficient used to account for differences between theoretical and actual yield (Guan et al., 2010; Gao et al., 2025).

### 2.5 Determination of nutrient content in rapeseed plants

The contents of total nitrogen (TN), total phosphorus (TP) and total potassium (TK) in rapeseed were determined at both the seedling stage and the flowering stage. In each treatment, five representative rapeseed plants were collected from each plot at both the seedling and flowering stages. Samples were initially oven-dried at 105 °C for 30 min, and subsequently dried at 75 °C to constant weight. Dried samples were then ground and passed through a 0.5-mm sieve. After digestion using the H_2_SO_4_–H_2_O_2_ method, TN was determined using a continuous flow analyzer, TP was measured using molybdenum-antimony absorbance spectrophotometry at 700 nm, and TK was determined using flame atomic absorption spectrophotometry.

### 2.6 Determination of soil physicochemical properties

Soil samples were collected from each plot under the three cropping patterns using the five-point sampling method. After air-drying at room temperature, they were sieved and sealed for storage, for subsequent analysis of soil physicochemical properties. Soil organic matter was determined using the dichromate oxidation method with external heating. Soil alkali-hydrolyzable nitrogen was determined using the alkaline hydrolysis diffusion method. Soil available phosphorus was determined using the molybdenum–antimony colorimetric method. Soil available potassium was determined by NH_4_OAc extraction followed by flame photometry. Soil pH and electrical conductivity were measured using a pH meter and an electrical conductivity meter, respectively.

### 2.7 Microbial community analysis

Total soil genomic DNA was extracted from soil samples using the FastPure Soil DNA Isolation Kit (Magnetic bead) (MJYH, Shanghai, China) according to the manufacturer’s instructions. DNA quality and integrity were assessed using agarose gel electrophoresis, and the DNA concentrations were quantified using a NanoDrop 2000 spectrophotometer (Thermo Fisher Scientific, Waltham, MA, USA). The bacterial 16S rRNA gene V3–V4 region was amplified using the primers 338F (5’-ACTCCTACGGGAGGCAGCAG-3’) and 806R (5’-GGACTACHVGGGTWTCTAAT-3’) (Bodenhausen et al., 2013), while the fungal ITS1 region was amplified using primers ITS1 (5′-TCCGTAGGTGAACCTGCGG-3′) and ITS2 (5′-GCTGCGTTCTTCATCGATGC-3′) (Zhang et al., 2010). PCR amplification was performed on a T100 Thermal Cycler (Bio-Rad, USA) using TransStart FastPfu DNA Polymerase (TransGen, AP221-02). PCR products were excised from 2% agarose gel and purified using the PCR Clean-Up Kit (YuHua, Shanghai, China) according to the manufacturer’s instructions and quantified using a Qubit 4.0 Fluorometer (Thermo Fisher Scientific, USA). Amplicons from each sample were pooled in equimolar concentrations to construct sequencing libraries, which were subsequently subjected to paired-end sequencing on an Illumina NextSeq 2000 platform (Illumina, San Diego, USA) following the standard protocols of Majorbio Bio-Pharm Technology Co., Ltd. (Shanghai, China). Raw reads were quality-filtered, denoised, merged, and chimera-checked using the DADA2 pipeline implemented in QIIME2, generating amplicon sequence variants (ASVs).

### 2.8 Data analysis

Data visualization was performed using GraphPad Prism 8 software, while statistical analyses were conducted using the SPSS 24.0 program. The significance of differences was assessed using Duncan’s new multiple range test at *P* < 0.05. Correlation analysis among soil physicochemical properties, plant nutrition, and crop theoretical yield was conducted using Pearson’s correlation coefficients. Low-abundance ASVs (<20 reads) were removed to minimize spurious signals and reduce noise in downstream analyses (Zhou et al., 2022). SparCC correlation coefficients among the retained ASVs were calculated using the OmicStudio platform (https://www.omicstudio.cn/tool/62) (Friedman and Alm, 2012). Significant associations were defined as correlations with an absolute coefficient value |r| > 0.8 and a significance level of *P* < 0.01 (Ye et al., 2023). Based on these thresholds, co-occurrence networks were constructed separately for bacterial and fungal communities. Network visualization and topological analysis were performed using Gephi software (version 0.10.1) to illustrate microbial interactions and overall network structure. Random forest analysis was performed using the “randomForest” package in R to identify discriminative microbial taxa among different cropping systems (Liu et al., 2025b). The model was applied to evaluate the importance of microbial taxa and to determine key contributors to community structure and associated ecological functions.

## 3 Results

### 3.1 Effects of different multiple cropping patterns on rapeseed yield components and theoretical yield

Rapeseed yield components and theoretical yield varied significantly among cropping patterns (**Table 1**). The number of seeds per pod was significantly higher in the tobacco–maize–rapeseed (T_M_R) and fallow–maize–rapeseed (F_M_R) treatments than in the tobacco–fallow–rapeseed (T_F_R) treatment. No significant differences in 1000-seed weight were observed among the treatments. Rapeseed theoretical yield with the addition of a maize season (T_M_R) reached 5388.48 kg/ha, which was significantly higher than that of the conventional T_F_R treatment, with no significant difference compared with the F_M_R treatment.

**Table 1:** Yield components and productivity of rapeseed under different multiple cropping systems.

Treatment	Density plant/hm^2^	Number of pods per plant	Number of seeds per pod	1000-seed weight/g	Yield Kg/ha
T_M_R	3.25×10^5^	214.58 ± 12.18a	24.8 ± 0.49a	3.59 ± 0.13a	5388.48 ± 231.76a
T_F_R	3.17×10^5^	187.92 ± 11.54a	22.27 ± 0.53b	3.82 ± 0.08a	4715.11 ± 106.61b
F_M_R	3.21×10^5^	204.08 ± 12.18a	24.69 ± 0.46a	3.80 ± 0.08a	5615.76 ± 204.01a

Data in the table as the mean ± standard error (n = 3). Different lowercase letters indicate significant differences among different multiple cropping systems based on one-way ANOVA followed by Duncan’s new multiple range test (*P* < 0.05). T_M_R, tobacco–rapeseed rotation with an additional relay maize crop; T_F_R, tobacco–rapeseed rotation; F_M_R, fallow–maize–rapeseed.

### 3.2 Effects of different multiple cropping patterns on the growth status of rapeseed

**Figure 1:**
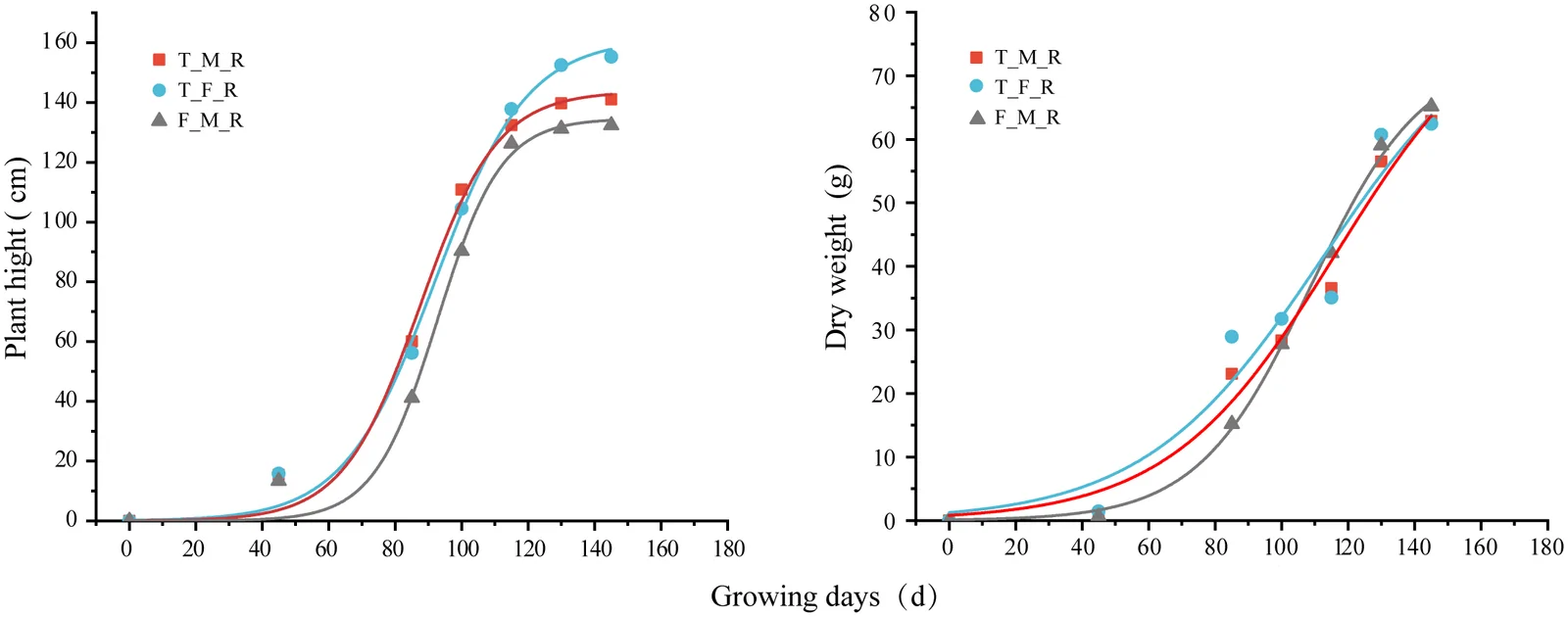
Logistic growth curves of plant height and dry weight of rapeseed under different cropping systems.

Different cropping patterns significantly affected rapeseed growth dynamics (**Figure 1**). Plant height under the T_M_R treatment was markedly higher than that under the F_M_R treatment. Similarly, at the early growth stage, rapeseed dry weight was greater under the T_M_R treatment than under the F_M_R treatment. However, these differences diminished at later growth stages. No significant differences in plant height or dry weight were observed between the T_M_R and T_F_R treatments throughout the growth period.

### 3.3 Effects of different multiple cropping patterns on logistic growth parameters of rapeseed

**Figure 2:**
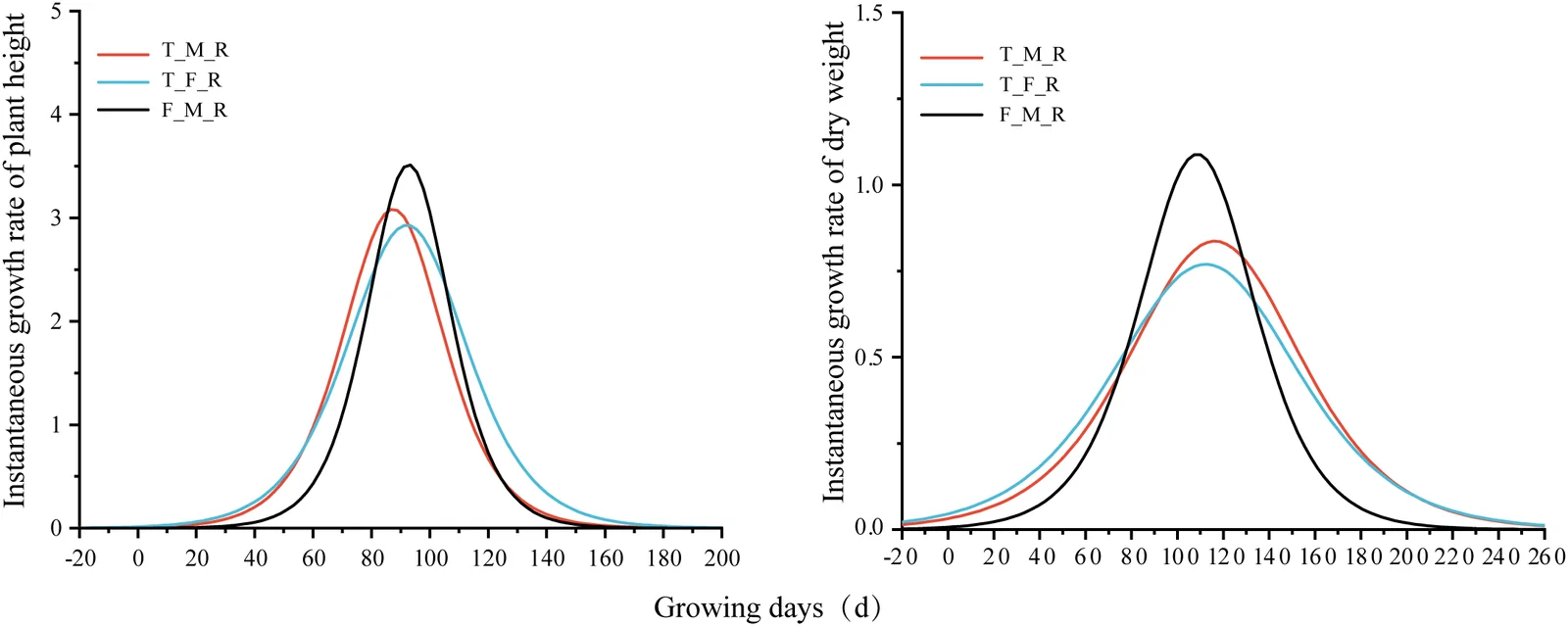
Instantaneous growth rate curves of plant height and dry weight of rapeseed under different cropping systems.

The logistic growth curve parameters of rapeseed plant height and dry weight differed among the different multiple cropping patterns (**Figure 2**, **Table 2**). Compared with the T_F_R pattern, the addition of a maize season (T_M_R) increased both the initial growth rate (*K*) and maximum instantaneous growth rate (*I_max_*) for plant height and dry weight. In contrast, compared with the F_M_R cropping pattern, prior tobacco planting increased the asymptotic maximum values (a) for both plant height and dry weight of rapeseed, while simultaneously decreasing the initial growth rate (*K*) and maximum instantaneous growth rate (*I_max_*).

**Table 2:** Estimated parameters of the logistic growth curves for rapeseed growth indices under different cropping systems.

Cropping patterns	Plant height				Dry weight			
	a	*K*/d^−1^	t50/d	*Imax*/d^−1^	a	*K*/d^−1^	t50/d	*Imax*/d^−1^
T_M_R	143.74 ± 5.68	0.086 ± 0.018	87.46 ± 2.03	3.09	83.95 ± 21.82	0.039 ± 0.011	116.29 ± 15.3	0.82
T_F_R	161.19 ± 6.67	0.073 ± 0.011	92.12 ± 2.02	2.94	82.78 ± 34.09	0.037 ± 0.018	112.36 ± 26.08	0.77
F_M_R	134.63 ± 5.24	0.104 ± 0.02	92.74 ± 1.79	3.5	73.52 ± 3.21	0.059 ± 0.005	108.65 ± 2.05	1.08

a. The asymptotic maximum value; K. the initial growth rate; t50.the time of reaching the maximum instantaneous rate, Imax. the maximum instantaneous growth rate. T_M_R, tobacco–rapeseed rotation with an additional relay maize crop; T_F_R, tobacco–rapeseed rotation; F_M_R, fallow–maize–rapeseed.

### 3.4 Effects of different multiple cropping patterns on the nutrition of rapeseed plants

**Figure 3:**
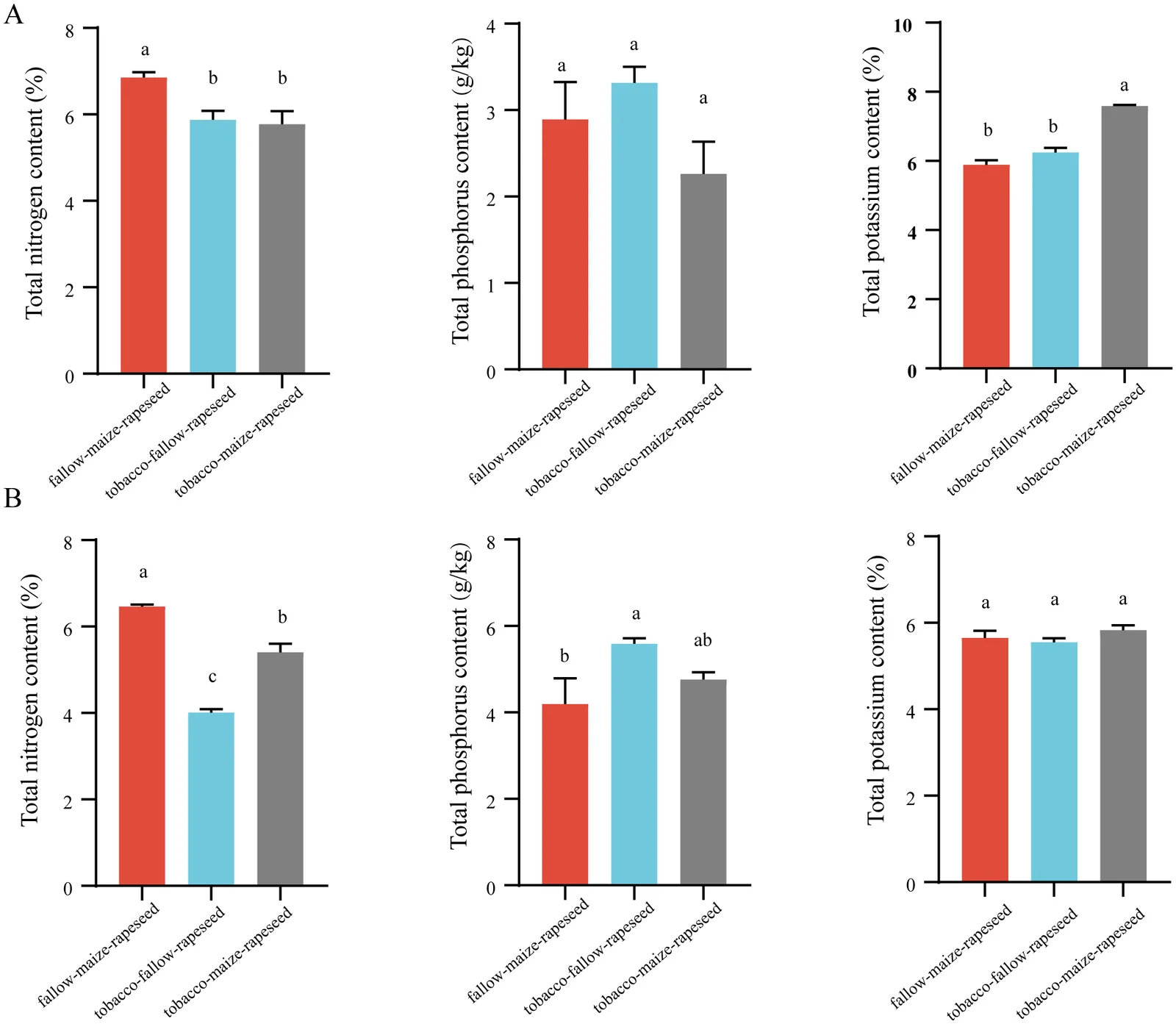
Effects of different cropping systems on nutrient contents in rapeseed plants. **(A)** TN, TP, and TK contents at the seedling stage of rapeseed; **(B)** TN, TP, and TK contents at the flowering stage. Error bars represent standard error (SE) of the mean (n = 3). Different lowercase letters indicate significant differences among treatments within the same growth stage based on one-way ANOVA followed by Duncan’s new multiple range test (*P* < 0.05). F_M_R, fallow–maize–rapeseed; T_F_R, tobacco–rapeseed rotation; T_M_R, tobacco–rapeseed rotation with an additional relay maize crop.

Different multiple cropping treatments had no significant effects on total nitrogen (TN) or total phosphorus (TP) contents at the seedling stage (**Figure 3A**). In contrast, total potassium (TK) content varied among treatments, with the highest value observed under the T_M_R treatment (7.59%) (**Figure 3A**). At the flowering stage, compared with the traditional T_F_R treatment, rapeseed TN content was significantly higher (*P* < 0.05) under the T_M_R treatment, but was lower than that in the F_M_R treatment (**Figure 3B**). The TP content of rapeseed under the T_M_R treatment was lower than that under the T_F_R treatment, but higher than that under the F_M_R treatment (**Figure 3B**). There were no significant differences in TK content among the three multiple cropping patterns (**Figure 3B**).

### 3.5 Analysis of soil physicochemical properties under different multiple cropping patterns

**Figure 4:**
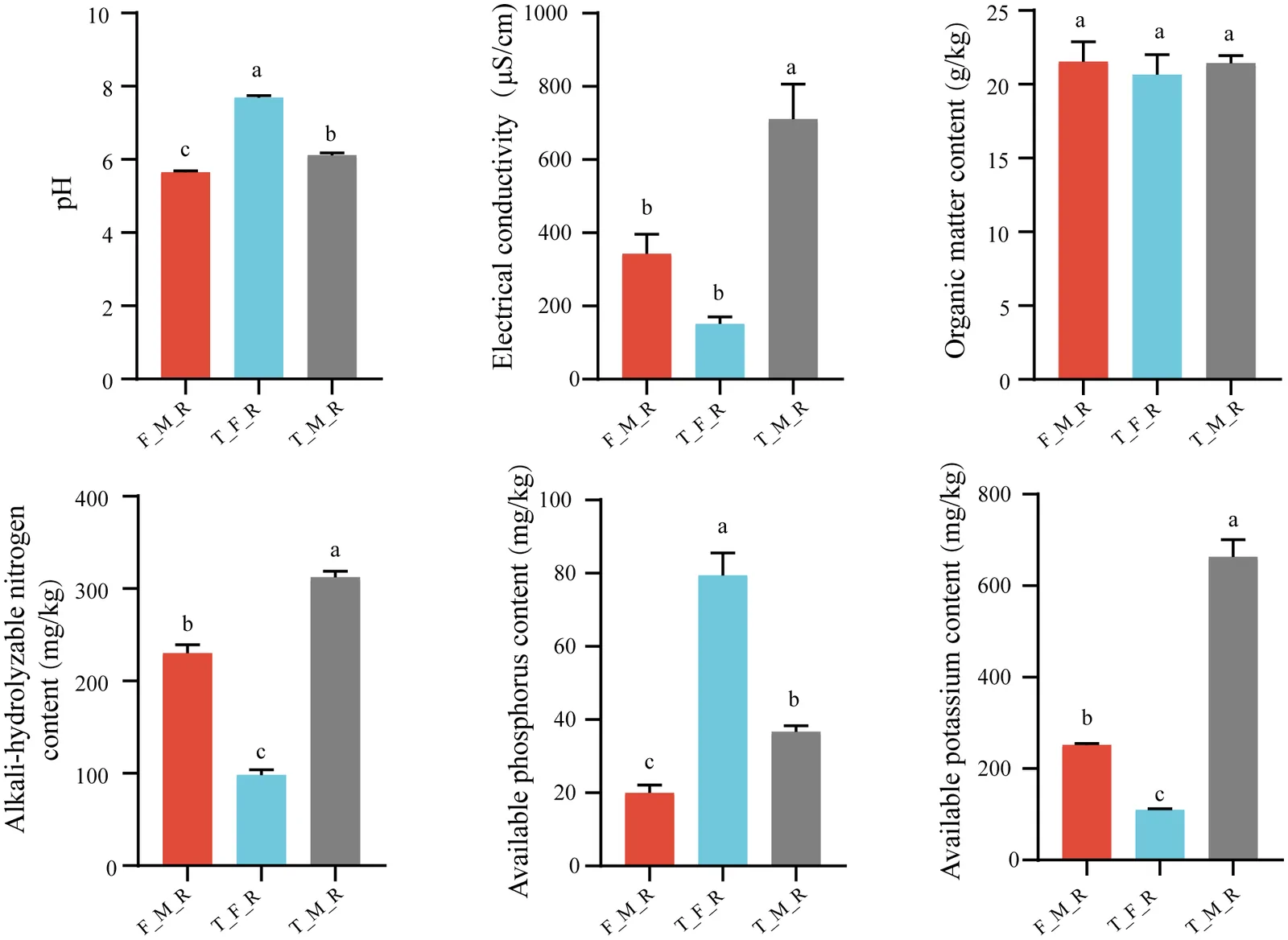
Soil physicochemical properties under different cropping systems before rapeseed planting. Error bars represent standard error (SE) of the mean (n = 3). Different letters indicate significant differences among treatments based on one-way ANOVA followed by Duncan’s new multiple range test (*P* < 0.05). F_M_R, fallow–maize–rapeseed; T_F_R, tobacco–rapeseed rotation; T_M_R, tobacco–rapeseed rotation with an additional relay maize crop.

Different multiple cropping patterns exerted significant effects on soil physicochemical properties (**Figure 4**). Before rapeseed planting, compared with the T_F_R treatment, the inclusion of an additional maize season (T_M_R) resulted in a decrease in soil pH and available phosphorus (AP) content, while electrical conductivity (EC), alkali-hydrolyzable nitrogen (AN), and available potassium (AK) were significantly increased (558.93 μS/cm, 214.03 mg/kg, and 553.66 mg/kg, respectively). In comparison with the F_M_R treatment, the T_M_R treatment showed higher soil pH, AP, EC, AN, and AK.

### 3.6 Relationships among rapeseed yield, soil properties, and plant nutrient status

**Figure 5:**
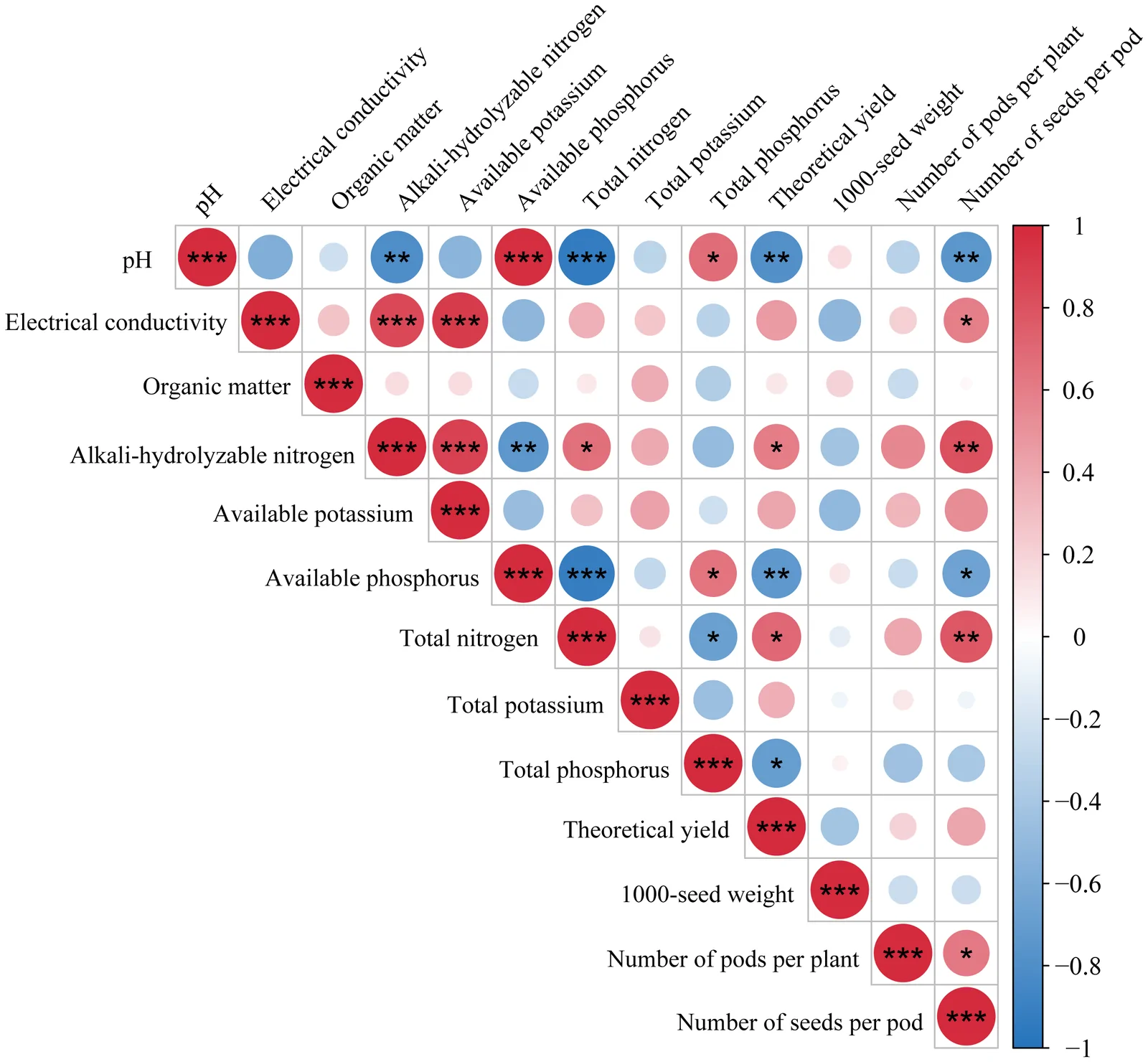
Correlation of soil physicochemical properties and plant nutrients with rapeseed theoretical yield and yield components. Correlation analyses were performed using Pearson’s correlation coefficient. The circle size represents the magnitude of the correlation coefficient; red indicates positive correlation, and blue indicates negative correlation. *, **, and *** denote significance at the levels of *P* < 0.05, *P* < 0.01, and *P* < 0.001, respectively.

Correlation analysis (**Figure 5**) revealed that rapeseed theoretical yield and seeds per pod were positively correlated with plant TN and soil AN, but negatively correlated with soil pH and AP. Regarding plant nutrient status, plant TN showed a significant positive correlation with soil AN, but a significant negative correlation with soil pH and AP. In contrast, plant TP showed positive correlations with soil pH and AP.

### 3.7 Effects of cropping patterns on soil microbial diversity and community structure

**Figure 6:**
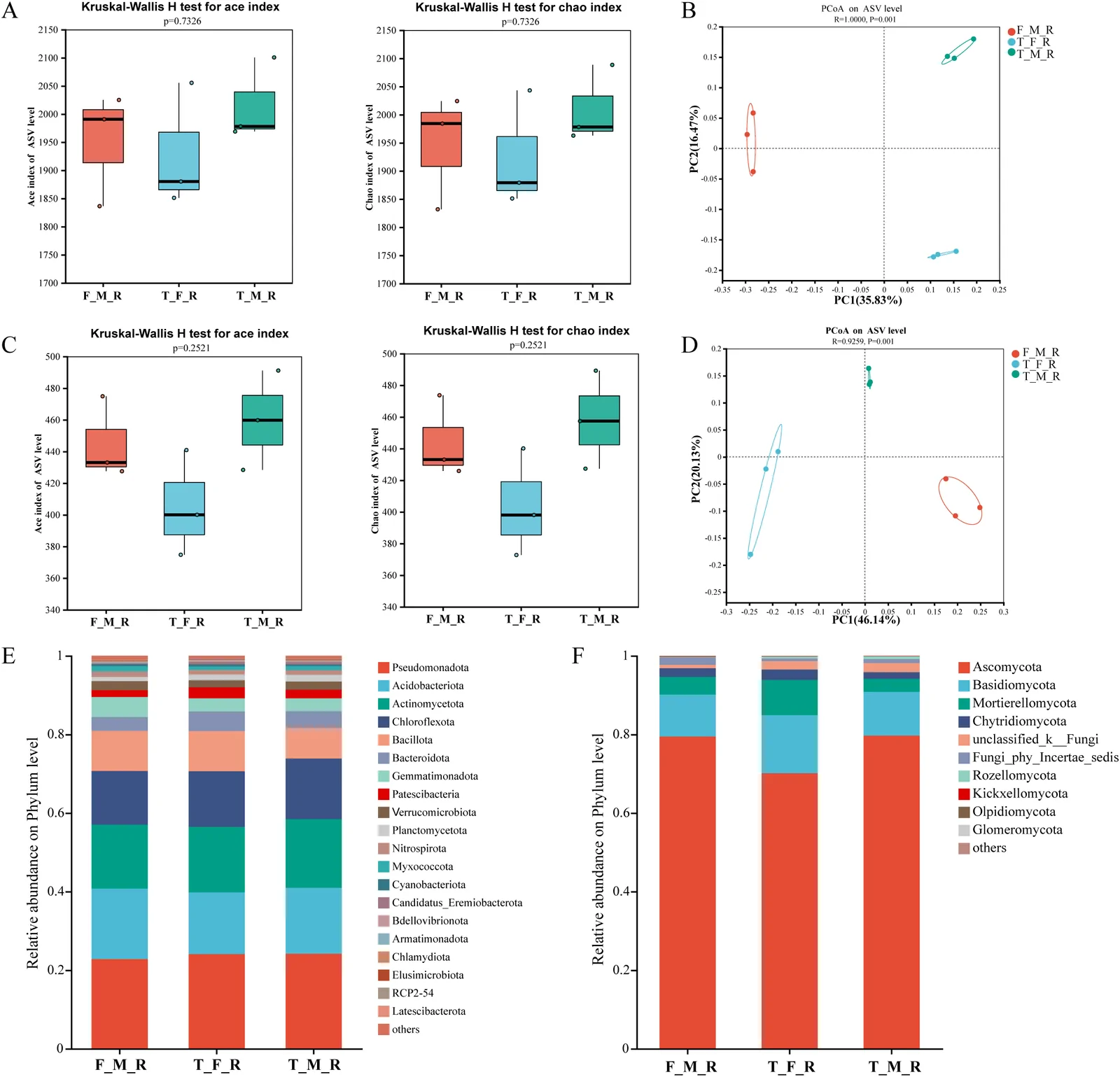
Differences in soil bacterial and fungal α-diversity, community structure, and phylum-level composition under different cropping systems. **(A–B)** Bacterial α-diversity (ACE and Chao1 indices) and β-diversity based on ASV-level data. **(C–D)** Fungal α-diversity (ACE and Chao1 indices) and β-diversity based on ASV-level data. E-F. Relative abundance of dominant bacterial and fungal phyla. α-diversity differences among treatments (ACE and Chao1 indices) were analyzed using the Kruskal–Wallis H test followed by Dunn’s multiple comparison test. Differences in bacterial and fungal community composition were evaluated using PCoA based on Bray–Curtis distances and tested by ANOSIM (999 permutations). F_M_R, fallow–maize–rapeseed; T_F_R, tobacco–rapeseed rotation; T_M_R, tobacco–rapeseed rotation with an additional relay maize crop.

No significant differences were observed in the α-diversity indices (ACE and Chao1) of bacterial and fungal communities among cropping patterns (*P* > 0.05; **Figures 6A, C**). However, compared with the conventional tobacco–rapeseed rotation (T_F_R), species richness tended to increase under the treatment with an additional season of maize cultivation (T_M_R) (**Figures 6A, C**). Principal coordinates analysis (PCoA) revealed clear separations in soil microbial community composition among cropping patterns at the ASV level. Significant differences in community composition were observed among treatments for bacterial and fungal communities (R = 1.00, *P* = 0.001; R = 0.93, *P* = 0.001) (**Figures 6B, D**). At the phylum level, bacterial communities were dominated by Pseudomonadota, Acidobacteriota, Actinomycetota, Chloroflexota, and Bacillota, with slight increases in the relative abundances of Acidobacteriota and Actinomycetota under the T_M_R treatment (**Figure 6E**). Fungal communities were dominated by Ascomycota, followed by Basidiomycota and Mortierellomycota. The relative abundance of Ascomycota varied among cropping systems, being lower under T_F_R and higher under F_M_R and T_M_R treatments (**Figure 6F**).

### 3.8 Changes in soil microbial co-occurrence network structure and topological properties under different cropping patterns

**Figure 7:**
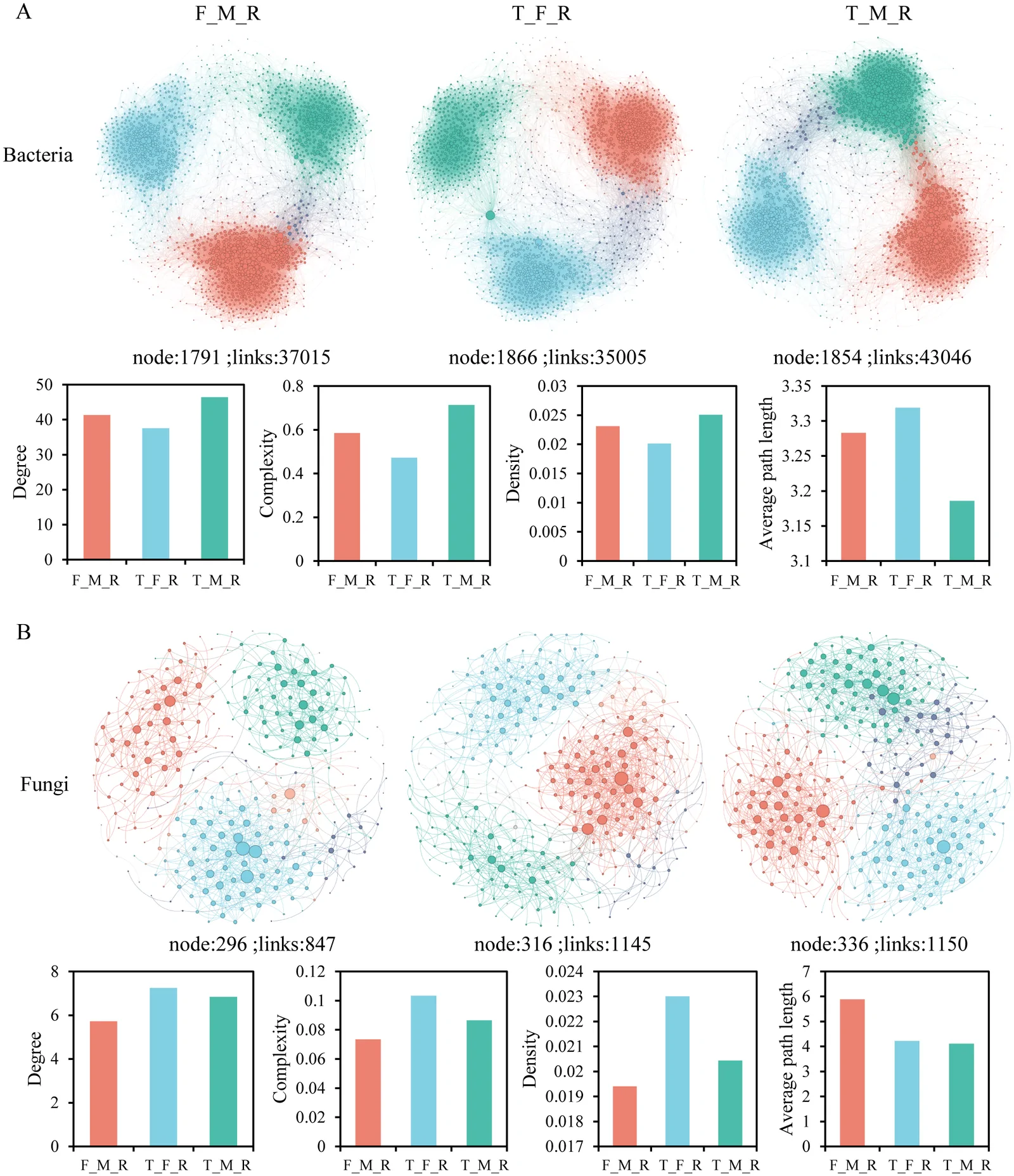
Comparison of co-occurrence network structure and topological properties of soil bacterial and fungal communities under different cropping systems. **(A)** Co-occurrence networks of bacterial communities and comparison of their topological parameters. **(B)** Co-occurrence networks of fungal communities and comparison of their topological parameters. Nodes represent microbial ASVs, and node size is proportional to degree. Different colors indicate distinct network modules. Edges represent significant correlations. F_M_R, fallow–maize–rapeseed; T_F_R, tobacco–rapeseed rotation; T_M_R, tobacco–rapeseed rotation with an additional relay maize crop.

Co-occurrence network analysis indicated that different cropping patterns significantly affected the structural characteristics of soil microbial communities (**Figure 7**). The bacterial network under relay maize cropping (T_M_R) contained a greater number of nodes and edges than those under the T_F_R and F_M_R treatments. Topological analysis further showed that T_M_R treatment had higher average degree, network complexity, and density, accompanied by a lower average path length (**Figure 7A**).

Compared with the bacterial community, the fungal networks exhibited fewer nodes and edges overall, but still displayed clear modular organization. Among the treatments, the T_M_R network contained the highest number of nodes. Topological parameter comparisons showed that the fungal networks under T_F_R and T_M_R treatments had higher average degree, network complexity, and density, accompanied by lower average path length (**Figure 7B**).

### 3.9 Abundance of dominant bacterial and fungal genera and identification of microbial biomarkers under different cropping patterns

**Figure 8:**
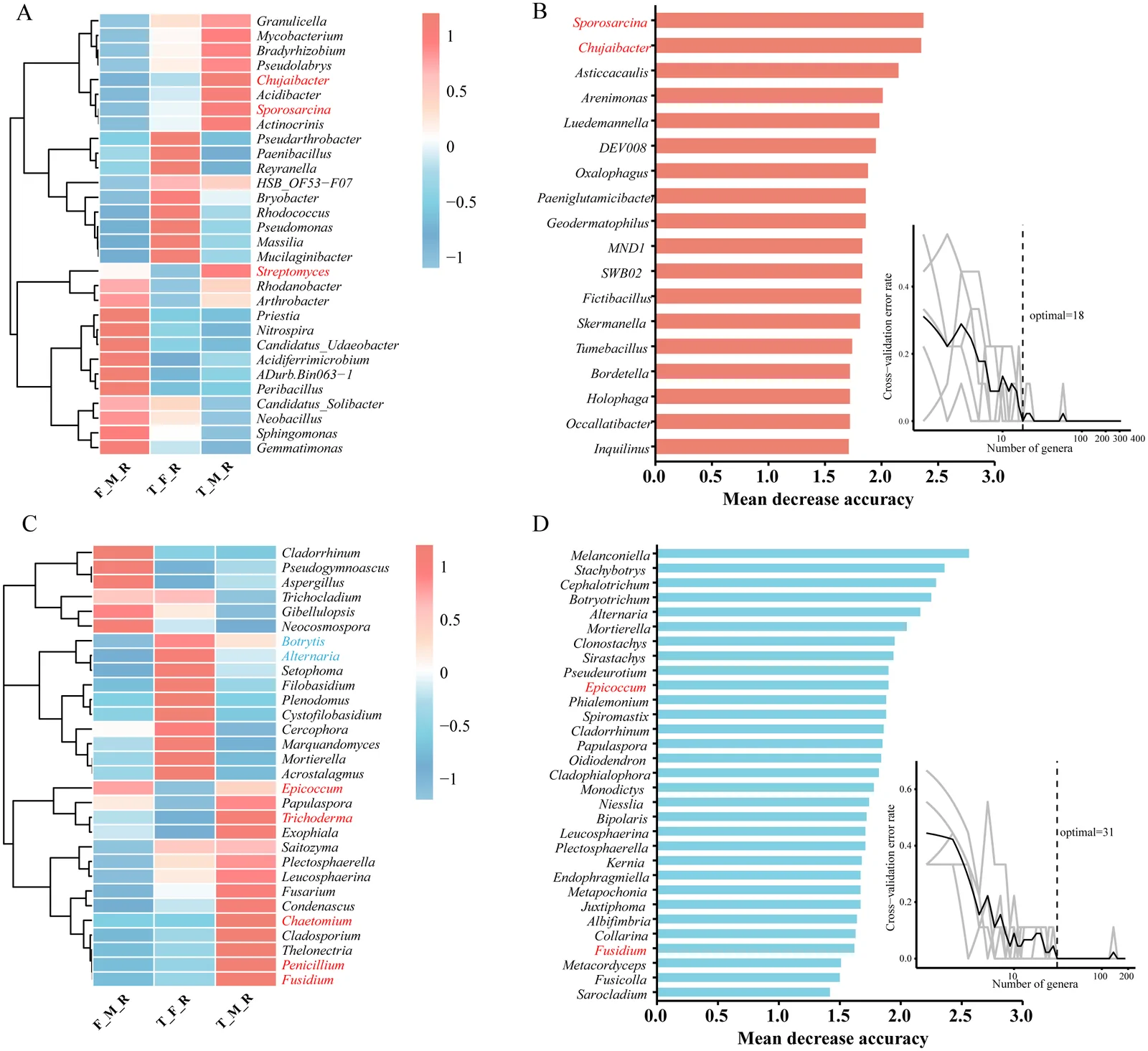
Abundance of dominant bacterial and fungal genera and identification of key microbial biomarkers under different cropping systems. **(A–C)** Heatmaps showing the relative abundances of the top 30 dominant bacterial **(A)** and fungal **(C)** genera under different cropping patterns (red indicates higher relative abundance in the tobacco–maize–rapeseed rotation, T_M_R; blue indicates lower abundance). **(B–D)** Microbial biomarkers identified by the random forest model and their contributions to classification accuracy. Insets show cross-validation error as a function of the number of feature genera, with the dashed line indicating the optimal number of features (bacteria: 18; fungi: 31). F_M_R, fallow–maize–rapeseed; T_F_R, tobacco–rapeseed rotation; T_M_R, tobacco–rapeseed rotation with an additional relay maize crop.

Differences were observed in the relative abundances of dominant bacterial and fungal genera among cropping patterns. In the bacterial community (**Figure 8A**), the relative abundances of *Chujaibacter*, *Sporosarcina*, and *Streptomyces* were higher under the relay maize cropping treatment (T_M_R), whereas these genera were comparatively less abundant in the T_F_R treatment. In the fungal community (**Figure 8C**), the T_M_R treatment showed higher abundances of *Epicoccum*, *Fusidium*, *Trichoderma*, and *Penicillium*, whereas the relative abundances of potential pathogenic fungi, including *Botrytis* and *Alternaria*, were reduced. Random forest analysis (**Figures 8B, D**) further identified key microbial biomarkers associated with cropping patterns. In the bacterial community, *Sporosarcina* and *Chujaibacter* were identified as the most important discriminatory genera. In the fungal community, *Epicoccum* and *Fusidium* were identified as key biomarkers distinguishing the T_M_R treatment.

## 4 Discussion

### 4.1 Constituent factors and yield changes of rapeseed under different multiple cropping patterns

Different multiple cropping patterns affect the yield components and final theoretical yield of rapeseed, highlighting the regulatory effect of preceding crop combinations on the growth, development, and yield formation of subsequent rapeseed (Sieling et al., 1997; Sieling and Christen, 2015). Compared with the traditional tobacco–rapeseed rotation (T_F_R), the inclusion of an additional season of maize (T_M_R) enhanced both land-use efficiency and rapeseed productivity. This effect may be partly attributed to maize root exudates and crop residues, which potentially improve soil structure and augment the availability of nutrients such as alkali-hydrolyzable nitrogen (AN) (Liu, 2024). Furthermore, although the F_M_R system is associated with lower economic value, rapeseed yield under the T_M_R treatment was comparable to that under the F_M_R system, suggesting that increased cropping intensity does not compromise overall productivity.

### 4.2 Effects of different multiple cropping patterns on the growth dynamics of rapeseed

Different multiple cropping patterns significantly influenced plant growth dynamics, which may be associated with nutrient accumulation, resource utilization efficiency, and soil environment enhancement (Tilman et al., 2002; Li et al., 2025b). The T_M_R cropping pattern exhibited distinct stage-dependent effects on rapeseed growth. During the early growth stage, the T_M_R treatment exhibited a higher initial growth rate (K) and maximum instantaneous growth rate (*I_max_*) for both plant height and dry weight compared with traditional T_F_R. This suggests that the T_M_R promotes early growth and enhances dry matter accumulation during the rapid growth stage, with the inclusion of maize contributing to an increased growth potential. However, during the late growth stage, rapeseed biomass accumulation tended to converge among the different multiple cropping treatments. Considering the significant yield increase observed under the T_M_R system, this suggests that once rapeseed growth enters a bottleneck phase, a greater proportion of photosynthates may be allocated to the seeds, and improved assimilate partitioning may contribute to yield formation (Diepenbrock, 2000). These findings provide a theoretical foundation for optimizing crop growth regulation and source-sink relationships in multiple cropping systems (Marcelis, 1996).

### 4.3 Regulatory effects of different multiple cropping patterns on nutrient absorption of rapeseed

This study suggests that preceding crop-based multiple cropping patterns influence the accumulation of nitrogen, phosphorus, and potassium nutrients during the seedling and flowering stages of rapeseed. This indicates that preceding crops may alter the nutrient uptake patterns of the subsequent rapeseed by modifying the soil nutrient environment or through interactions among crop roots (Town et al., 2023; Wang et al., 2023b; Malik et al., 2025). Specifically, the cultivation of tobacco or maize as the preceding crop enhances potassium uptake in rapeseed seedlings. This effect may be related to the increased availability of potassium in the soil following the cultivation of these crops (Xu et al., 2025; Zhao et al., 2025a). Tobacco and maize differ from other crops in their potassium uptake and allocation characteristics during growth. After decomposition of their residues in the soil, soluble potassium is released thereby increasing soil available potassium and providing sufficient potassium for the early growth of subsequent rapeseed (Niu, 2013). The results obtained in this study are consistent with previous findings. Soil available potassium content in rapeseed fields under the T_M_R system was significantly higher than that under other multiple cropping patterns, showing increases of 553.66 and 411.47 mg/kg compared with the T_F_R and F_M_R systems, respectively. Furthermore, from the perspective of nutrient accumulation during the flowering period of rapeseed, the cultivation of maize as a preceding crop can enhance nitrogen absorption in rapeseed. The substantial increase in alkali-hydrolyzable nitrogen in the soil associated with the T_M_R pattern may be a critical factor contributing to this enhancement (McDaniel and Grandy, 2016). Additionally, the regulation of soil microbial communities, soil enzyme activities, and the decomposition rate of organic matter by the preceding crop may also contribute to differences in nutrient uptake of rapeseed across various multiple cropping patterns (Chen et al., 2019; Zhao et al., 2025b). Therefore, understanding the interrelationships among the preceding crop, soil environment, and subsequent crop nutrition is important for optimizing multiple cropping patterns and improving nutrient utilization efficiency.

### 4.4 Changes in soil physicochemical properties under multiple cropping systems and their key influencing factors on yield

Alterations in soil physicochemical properties induced by different multiple cropping patterns may reflect the effects of cropping system variation (Gikonyo et al., 2022; Liang et al., 2023). Previous studies have demonstrated that multiple cropping systems can improve soil physicochemical properties (Wu et al., 2024; Wang et al., 2023a). In this study, soil pH and available phosphorus (AP) responded differently to preceding cropping systems, whereas electrical conductivity (EC), alkali-hydrolyzable nitrogen (AN), and available potassium (AK) were consistently higher in T_M_R treatment than under T_F_R and F_M_R. Among the treatments, the T_M_R treatment exhibited the most pronounced effect on improving soil physical and chemical properties, indicating the combined effects of tobacco and maize residues on soil nutrients. Notably, maize has high phosphorus demand during growth, whereas tobacco may promote phosphorus availability. This phenomenon may be attributed to maize’s high phosphorus requirements and the capacity of tobacco roots to mobilize soil phosphorus. The positive correlations of rapeseed theoretical yield and seeds per pod with plant total nitrogen and soil alkali-hydrolyzable nitrogen highlight the critical role of nitrogen availability in yield formation. Adequate nitrogen supply can enhance plant growth and assimilate production, thereby promoting seed formation and increasing yield potential (Tang et al., 2012; Zhao et al., 2016). Conversely, soil available phosphorus and pH were negatively correlated with yield-related traits. This relationship may reflect the influence of soil chemical conditions on nutrient availability and plant nutrient uptake. Previous studies have reported that excessive phosphorus availability can induce nutrient imbalances, thereby inhibiting the uptake of other essential nutrients and potentially reducing crop yield (Xu et al., 2014). In summary, soil alkali-hydrolyzable nitrogen and plant total nitrogen are critical factors influencing rapeseed yield components. These results support our first hypothesis that improved nitrogen availability under the T_M_R system promotes rapeseed growth and productivity.

### 4.5 Cropping patterns influence system productivity by shaping soil microbial community structure

The results of this study indicate that the T_M_R triple-cropping system significantly optimizes the structure and interaction networks of the soil microbial community, which may contribute to the improved productivity observed in this system. Compared with the conventional T_F_R system, the insertion of a relay maize crop (T_M_R) did not significantly alter the α-diversity (ACE and Chao1 indices) of bacterial and fungal communities, suggesting that microbial richness remained relatively stable (Wagg et al., 2019). However, β-diversity analysis (PCoA) revealed a distinct separation, indicating substantial shifts in community composition likely driven by differences in root exudates and residue inputs associated with crop sequences (Vukicevich et al., 2016). Importantly, the additional season of maize cultivation (T_M_R) induced a more complex and interconnected bacterial co-occurrence network. These topological characteristics indicate greater network connectivity and complexity, which may enhance microbial interactions and nutrient cycling processes (de Vries et al., 2018; Ye et al., 2022). This suggests that cropping system intensification may regulate microbial interactions rather than simply altering species richness. The additional season of maize cultivation (T_M_R) enriched bacterial taxa such as *Chujaibacter*, *Sporosarcina*, and *Streptomyces*, which are commonly involved in organic matter decomposition, nitrogen transformation, and disease suppression (Bhattacharyya and Jha, 2012). The importance of *Sporosarcina* and *Chujaibacter* in the random forest model further highlights their potential role as key biomarkers in shaping microbial responses to cropping systems. Their enrichment may be driven by increased inputs of root-derived organic carbon, including maize root residues and rhizodeposits (Paterson et al., 2007; Marschner et al., 2012). In the fungal community, key genera including *Epicoccum*, *Fusidium*, *Trichoderma*, and *Penicillium* were enriched under the T_M_R treatment, while potential pathogenic genera such as *Botrytis* and *Alternaria* were reduced. These beneficial fungi are well documented to exhibit antagonistic activity against soilborne pathogens and to promote plant growth (Mazzola and Freilich, 2017; Feng et al., 2022). *Fusidium* can produce the bioactive secondary metabolite fusidic acid, but its ecological functions in soil remain to be further investigated (Huang et al., 2021). Therefore, the T_M_R system appears to reshape soil microbial community structure by integrating sequential crop effects from tobacco and maize, thereby promoting microbial assemblages enriched in functional microorganisms associated with nutrient cycling and plant health. This shift in microbial community composition and network structure was associated with improved soil nitrogen availability, enhanced rapeseed nitrogen nutrition, and increased system productivity (Fierer, 2017). These results are consistent with our second hypothesis, suggesting that cropping system intensification can regulate soil microbial community assembly and functional potential. However, as this study is based on field observations and correlation analyses, further experimental validation is required to confirm the functional roles of key microbial taxa in nutrient cycling and crop productivity.

## 5 Conclusion

This study demonstrates that the tobacco–maize–rapeseed triple-cropping system enhances rapeseed productivity through optimized crop sequencing and improved nutrient availability and uptake. Compared with the conventional tobacco–fallow–rapeseed rotation, the inclusion of a maize season increased theoretical yield and yield components while promoting early growth vigor and nutrient accumulation, particularly nitrogen availability and uptake. Soil alkali-hydrolyzable nitrogen and plant total nitrogen were identified as key determinants of yield formation. The triple-cropping system also reshaped soil microbial communities, increasing bacterial co-occurrence network complexity. *Chujaibacter*, *Sporosarcina*, and *Epicoccum* were among the taxa enriched under the triple-cropping system and were associated with improved nutrient status and higher yield. Overall, this integrated cropping strategy provides a promising approach for sustainable cropping intensification in diversified agricultural systems.

## Data Availability

The original contributions presented in this study are included in the article. Raw soil microbiome amplicon sequencing data have been deposited in the NCBI Sequence Read Archive (SRA) under accession number PRJNA1430241.

## Supplementary material

Supplementary Table 1 Initial physicochemical properties of the experimental soil.

## References

[B1] Bhattacharyya P. N. Jha D. K. (2012). Plant growth-promoting rhizobacteria (PGPR): emergence in agriculture. World J. Microbiol. Biotechnol. 28, 1327–1350. doi: 10.1007/s11274-011-0979-9 22805914

[B2] Bodenhausen N. Horton M. W. Bergelson J. (2013). Bacterial communities associated with the leaves and the roots of Arabidopsis thaliana. PloS One 8, e56329. doi: 10.1371/journal.pone.0056329 23457551 PMC3574144

[B3] Chen H. Wei L. Wang Y. Huang J. Zhao J. Zhang C. (2019). Effects of different planting and fertilizing modes on soil nutrient, enzyme activity and bacterial diversity of tobacco. J. South. Agric. 50, 982–989. doi: 10.3969/j.issn.2095-1191.2019.05.09

[B4] Deng J. Zhang Y. Hu J. Jiao J. Hu F. Li H. . (2017). Autotoxicity of phthalate esters in tobacco root exudates: effects on seed germination and seedling growth. Pedosphere 27, 1073–1082. doi: 10.1016/S1002-0160(17)60374-6

[B5] de Vries F. T. Griffiths R. I. Bailey M. Craig H. Girlanda M. Gweon H. S. . (2018). Soil bacterial networks are less stable under drought than fungal networks. Nat. Commun. 9, 3033. doi: 10.1038/s41467-018-05516-7 30072764 PMC6072794

[B6] Diepenbrock W. (2000). Yield analysis of winter oilseed rape (Brassica napus L.): a review. Field Crops Res. 67, 35–49. doi: 10.1016/S0378-4290(00)00082-4

[B7] Dong M. Zhang Q. Wang Y. Wang S. Feng G. Liang Q. . (2024). Broadband crop rotation of cotton-grain-rape improved crop yield and light utilization efficiency. Chin. J. Eco-Agriculture 32, 1159–1169. doi: 10.12357/cjea.20230753

[B8] Fang Y. Zhang L. Jiao Y. Liao J. Luo L. Ji S. . (2016). Tobacco rotated with rapeseed for soil-borne phytophthora pathogen biocontrol: mediated by rapeseed root exudates. Front. Microbiol. 7, 894. doi: 10.3389/fmicb.2016.00894 27379037 PMC4904020

[B9] Feng N. Li Q. Mu Q. Lin S. Liu S. Li H. . (2022). Metabolites and antifungal activities of an endophytic fungus Epicoccum sorghinum from mangrove. J. South. China Agric. Univ. 43, 77–81. doi: 10.7671/j.issn.1001-411X.202112017

[B10] Fierer N. (2017). Embracing the unknown: disentangling the complexities of the soil microbiome. Nat. Rev. Microbiol. 15, 579–590. doi: 10.1038/nrmicro.2017.87 28824177

[B11] Friedman J. Alm E. J. (2012). Inferring correlation networks from genomic survey data. PloS Comput. Biol. 8, e1002687. doi: 10.1371/journal.pcbi.1002687 23028285 PMC3447976

[B12] Gałązka A. Gawryjołek K. Perzyński A. Gałązka R. Jerzy K. (2017). Changes in enzymatic activities and microbial communities in soil under long-term maize monoculture and crop rotation. Pol. J. Environ. Stud. 26, 39–46. doi: 10.15244/pjoes/6474539883939

[B13] Gao Y. Zhang S. Wang J. Wang K. Zhang J. Lu J. . (2025). Fertilization pattern and nitrogen fertilizer operation on yield and quality of rapeseed with dual-use of vegetable and oil in the Erhai Lake Basin. Chin. J. Oil Crop Sci. 47, 754–767. doi: 10.19802/j.issn.1007-9084.2024250

[B14] Gikonyo F. N. Dong X. Mosongo P. S. Guo K. Liu X. (2022). Long-term impacts of different cropping patterns on soil physico-chemical properties and enzyme activities in the low land plain of north China. Agronomy 12, 471. doi: 10.3390/agronomy1202047130654563

[B15] Gill K. S. (2018). Crop rotations compared with continuous canola and wheat for crop production and fertilizer use over 6 yr. Can. J. Plant Sci. 98, 1139–1149. doi: 10.1139/cjps-2017-029234819996

[B16] Guan C. Tang T. Wang G. He J. (2010). Comparison of four yield estimating methods in rapeseed field. Chin. J. Oil Crop Sci. 32, 187–190.

[B17] Hirzel J. Undurraga P. León L. Matus I. (2023). Effect of three crop rotations and four residue levels on canola and bean grain yield and residue production. J. Agric. Sci. 161, 272–278. doi: 10.1017/S002185962300016341292463

[B18] Huang W. W. Ge X. Y. Huang Y. Chai X. T. Zhang L. Zhang Y. X. . (2021). High-yield strain of fusidic acid obtained by atmospheric and room temperature plasma mutagenesis and the transcriptional changes involved in improving its production in fungus Fusidium coccineum. J. Appl. Microbiol. 130, 405–415. doi: 10.1111/jam.14797 32734700

[B19] Jiang Y. Liang Y. Li C. Wang F. Sui Y. Suvannang N. . (2016). Crop rotations alter bacterial and fungal diversity in paddy soils across East Asia. Soil Biol. Biochem. 95, 251–261. doi: 10.1016/j.soilbio.2016.01.00738826717

[B20] Li C. Hoffland E. Kuyper T. W. Yu Y. Zhang C. Li H. . (2020). Syndromes of production in intercropping impact yield gains. Nat. Plants 6, 653–660. doi: 10.1038/s41477-020-0680-9 32483328

[B21] Li H. Li C. Song X. Liu Y. Gao Q. Zheng R. . (2022). Impacts of continuous and rotational cropping practices on soil chemical properties and microbial communities during peanut cultivation. Sci. Rep. 12, 2758. doi: 10.1038/s41598-022-06789-1 35177784 PMC8854431

[B22] Li C. Shi L. Wang K. Liu B. Liao J. An Z. . (2025a). Crop rotation differentially increases soil bacterial and fungal diversities in global croplands: a meta-analysis. Nat. Commun. 16, 11686. doi: 10.1038/s41467-025-66823-4 41309652 PMC12749399

[B23] Li W. Sun J. Li C. Zhao J. Tang Y. Wu G. (2025b). Dynamic simulation and analysis of crop growth curves in wheat and faba bean intercropping system. J. Cold-Arid Agric. Sci. 4, 47–52. doi: 10.3969/j.issn.2097-2172.2025.01.008

[B24] Li J. Yan J. Hu W. Li X. Cong R. Ren T. . (2019). Effects of combined application of nitrogen and potassium on seed yield and nitrogen utilization of winter oilseed rape (Brassica napus L.). Acta Agron. Sin. 45, 941–948. doi: 10.3724/sp.j.1006.2019.84146

[B25] Li S. Yu S. Duan Z. Fu M. (2014). Cultivation technology of “Tobacco—Maize—Rapeseed” and its application in Tengchong county, Yunnan province. Crop Res. 28, 419–421. doi: 10.3969/j.issn.1001-5280.2014.04.21

[B26] Liang Y. Dai X. Wu K. Yang S. Zhao J. Dai Z. . (2023). Effects of millet-rape multi-cropping system on soil physical and chemical properties and microbial quantity in the hilly area. Guizhou Agric. Sci. 51, 46–52. doi: 10.3969/i.issn.1001-3601.2023.12.007

[B27] Ling N. Wang T. Kuzyakov Y. (2022). Rhizosphere bacteriome structure and functions. Nat. Commun. 13, 836. doi: 10.1038/s41467-022-28448-9 35149704 PMC8837802

[B28] Liu B. (2024). Study on decomposition characteristics of maize plant residues from different parts and its effect on soil fertility. Changchun: Jilin Agricultural University.

[B29] Liu L. Gao Y. Gao Z. Zhu L. Yan R. Yang W. . (2023). The core microbiota as a predictor of soil functional traits promotes soil nutrient cycling and wheat production in dryland farming. Funct. Ecol. 37, 2325–2337. doi: 10.1111/1365-2435.1438840046247

[B30] Liu Y. Han Q. Zhang J. Zhang X. Chen Y. Li M. . (2025a). Soybean nodulation shapes the rhizosphere microbiome to increase rapeseed yield. J. Adv. Res. 75, 95–110. doi: 10.1016/j.jare.2024.11.034 39674502 PMC12536662

[B31] Liu Y. Lu R. Tian G. Li X. Zhao S. Luo L. . (2025b). Root-secreted saponins weaken soil disease suppression ability by shaping rhizosphere microbial communities in Panax notoginseng. Microbiol. Res. 299, 128263. doi: 10.1016/j.micres.2025.128263 40582084

[B32] Liu B. Zou X. Song L. Guan Y. (2017). Comparative studies on crop yield, profit, nitrogen absorption and soil nutrient dynamics in paddy fields under different crop rotation systems. Acta Agric. Jiangxi 29, 1–7, 31. doi: 10.19386/j.cnki.jxnyxb.2017.12.01

[B33] Malik A. I. Bell R. Zang H. Boitt G. Whalley W. R. (2025). Exploring the plant and soil mechanisms by which crop rotations benefit farming systems. Plant Soil 507, 1–9. doi: 10.1007/s11104-024-06994-z30311153

[B34] Marcelis L. F. M. (1996). Sink strength as a determinant of dry matter partitioning in the whole plant. J. Exp. Bot. 47, 1281–1291. doi: 10.1093/jxb/47.Special_Issue.1281 21245260

[B35] Marschner P. Marhan S. Kandeler E. (2012). Microscale distribution and function of soil microorganisms in the interface between rhizosphere and detritusphere. Soil Biol. Biochem. 49, 174–183. doi: 10.1016/j.soilbio.2012.01.03338826717

[B36] Mazzola M. Freilich S. (2017). Prospects for biological soilborne disease control: application of indigenous versus synthetic microbiomes. Phytopathology 107, 256–263. doi: 10.1094/PHYTO-09-16-0330-RVW 27898265

[B37] McDaniel M. D. Grandy A. S. (2016). Soil microbial biomass and function are altered by 12 years of crop rotation. Soil 2, 583–599. doi: 10.5194/soil-2-583-2016

[B38] Meng J. Xie Z. Liu T. Chen G. Li Y. (2023). High-yield cultivation techniques for post-tobacco maize in Baoshan City. China Agric. Technol. Extension 39, 47–49. doi: 10.3969/j.issn.1002-381X.2023.03.015

[B39] Niu L. (2013). Influence research on tobacco residues decomposition, soil nutrients, enzyme activities and microorganism. Hunan Agricultural University: Changsha.

[B40] Paterson E. Gebbing T. Abel C. Sim A. Telfer G. (2007). Rhizodeposition shapes rhizosphere microbial community structure in organic soil. New Phytol. 173, 600–610. doi: 10.1111/j.1469-8137.2006.01931.x 17244055

[B41] Ruan Z. Zhou J. Li H. Wang Q. Fu J. Ji Y. . (2024). Effects of nitrogen fertilizer topdressing on nitrogen utilization, yield, and quality of early maturing maize intercropping with tobacco. J. Yunnan Agric. Univ. (Natural Science) 39, 151–159. doi: 10.12101/j.issn.1004-390X(n).202312015

[B42] Savary S. Ficke A. Aubertot J.-N. Hollier C. (2012). Crop losses due to diseases and their implications for global food production losses and food security. Food Secur. 4, 519–537. doi: 10.1007/s12571-012-0200-530311153

[B43] Sieling K. Christen O. (2015). Crop rotation effects on yield of oilseed rape, wheat and barley and residual effects on the subsequent wheat. Arch. Agron. Soil Sci. 61, 1531–1549. doi: 10.1080/03650340.2015.101756937339054

[B44] Sieling K. Christen O. Nemati B. Hanus H. (1997). Effects of previous cropping on seed yield and yield components of oil-seed rape (brassica napus L.). Eur. J. Agron. 6, 215–223. doi: 10.1016/S1161-0301(96)02049-7

[B45] Tang Y. Zuo Q. Leng S. Liu R. Ge Y. Jin C. . (2012). Effects of nitrogen absorption and utilization on yield formation of direct-sowing rapeseed in paddy rice field under different nitrogen application levels. Guangdong Agric. Sci. 39, 4–6. doi: 10.16768/j.issn.1004-874x.2012.10.046

[B46] Tian X. Duan J. Huo H. Huangfu J. Yan M. Lu H. . (2025). Effects of different crop rotations on microbial diversity and enzyme activities in Brassica napus rhizosphere soil. Microorganisms 14 (1), 91. doi: 10.3390/microorganisms14010091 41597610 PMC12844264

[B47] Tilman D. Cassman K. G. Matson P. A. Naylor R. Polasky S. (2002). Agricultural sustainability and intensive production practices. Nature 418, 671–677. doi: 10.1038/nature01014 12167873

[B48] Town J. R. Dumonceaux T. Tidemann B. Helgason B. L. (2023). Crop rotation significantly influences the composition of soil, rhizosphere, and root microbiota in canola (Brassica napus L.). Environ. Microbiome 18, 40. doi: 10.1186/s40793-023-00495-9 37161618 PMC10169384

[B49] Trinder C. Brooker R. Davidson H. Robinson D. (2012). Dynamic trajectories of growth and nitrogen capture by competing plants. New Phytol. 193, 948–958. doi: 10.1111/j.1469-8137.2011.04020.x 22236094

[B50] Vukicevich E. Lowery T. Bowen P. Úrbez-Torres J. R. Hart M. (2016). Cover crops to increase soil microbial diversity and mitigate decline in perennial agriculture. a review. Agron. Sustain. Dev. 36, 48. doi: 10.1007/s13593-016-0385-730311153

[B51] Wagg C. Schlaeppi K. Banerjee S. Kuramae E. E. van der Heijden M. G. A. (2019). Fungal-bacterial diversity and microbiome complexity predict ecosystem functioning. Nat. Commun. 10, 4841. doi: 10.1038/s41467-019-12798-y 31649246 PMC6813331

[B52] Wang Z. Dan Y. (2015). Exploration of the 'Tobacco + Grain + Oil' three-crop-a-year model. Yunnan Agric. Sci. Technol. (4), 32–34. doi: 10.3969/j.issn.1000-0488.2015.04.013

[B53] Wang Y. Niu F. Wang G. Zhao W. Yu D. Sun L. . (2026). Effects of crop rotation and continuous cropping on soil microbial community structure at different stages of tobacco. Plant Growth Regul. 106, 11. doi: 10.1007/s10725-025-01422-330311153

[B54] Wang Y. Shi M. Zhang R. Zhang W. Liu Y. Sun D. . (2023a). Legume-potato rotation affects soil physicochemical properties, enzyme activity, and rhizosphere metabolism in continuous potato cropping. Chem. Biol. Technol. Agric. 10, 132. doi: 10.1186/s40538-023-00508-238164791

[B55] Wang Y. Zhang H. Zhang Y. Fei J. Xiangmin R. Peng J. . (2023b). Crop rotation-driven changes in rhizosphere metabolite profiles regulate soil microbial diversity and functional capacity. Agric. Ecosyst. Environ. 358, 108716. doi: 10.1016/j.agee.2023.10871638826717

[B56] Wei H. Gong T. Zhou L. Qin L. (2026). Synergistic improvement in wheat yield, water and nitrogen use efficiency in wheat–maize rotation systems: A meta-analysis of multidimensional agricultural practices. Plants 15 (4), 617. doi: 10.3390/plants15040617 41754323 PMC12944248

[B57] Wu T. Gu G. Zhang B. Zhang Z. Chi G. Wang R. . (2024). Physicochemical properties and microbial community of soil and crop yield under rice-tobacco-milk vetch rotation cropping. Fujian J. Agric. Sci. 39, 984–992. doi: 10.19303/j.issn.1008-0384.2024.08.012

[B58] Xu X. He P. Pampolino M. F. Johnston A. M. Qiu S. Zhao S. . (2014). Fertilizer recommendation for maize in China based on yield response and agronomic efficiency. Field Crops Res. 157, 27–34. doi: 10.1016/j.fcr.2013.12.01338826717

[B59] Xu Z. Li C. Zhang C. Yu Y. van der Werf W. Zhang F. (2020). Intercropping maize and soybean increases efficiency of land and fertilizer nitrogen use; a meta-analysis. Field Crops Res. 246, 107661. doi: 10.1016/j.fcr.2019.10766138826717

[B60] Xu J. Yao Y. Pan L. Zhang N. Li D. Chen X. (2025). Pea–cucumber crop rotation suppresses fusarium pathogens by reshaping soil microbial communities and enhancing nutrient availability. Front. Microbiol. 16, 1697343. doi: 10.3389/fmicb.2025.1697343 41311484 PMC12647031

[B61] Yang J. Zhang S. Zhang J. Zhao S. Lu H. Li L. . (2025). Incorporating crop rotation into the winter wheat-summer maize system to enhance soil multifunctionality and sustainable grain production in the north China plain. Field Crops Res. 325, 109834. doi: 10.1016/j.fcr.2025.10983438826717

[B62] Ye C. Gong Y. Chen M. Delgado-Baquerizo M. Che R. Liu S. . (2023). Revegetation promotes soil microbial network stability in a novel riparian ecosystem. J. Appl. Ecol. 60, 1572–1586. doi: 10.1111/1365-2664.1444940046247

[B63] Ye Z. Wang J. Li J. Liu G. Dong Q. Zou Y. . (2022). Different roles of core and noncore bacterial taxa in maintaining soil multinutrient cycling and microbial network stability in arid fertigation agroecosystems. J. Appl. Ecol. 59, 2154–2165. doi: 10.1111/1365-2664.1422840046247

[B64] Zhang Y. (2020). Effect of chemical fertilizer reduction on rape yielding and benefit under rice straw returning. Fujian Sci. Technol. Rice Wheat 38, 30–32. doi: 10.3969/j.issn.1008-9799.2020.03.015

[B65] Zhang Y. J. Zhang S. Liu X. Z. Wen H. A. Wang M. (2010). A simple method of genomic DNA extraction suitable for analysis of bulk fungal strains. Lett. Appl. Microbiol. 51, 114–118. doi: 10.1111/j.1472-765X.2010.02867.x 20536704

[B66] Zhao C. Li Y. Liu Y. Wang X. Zhao W. Huang Y. . (2025b). Effects of rotating cropping and continuous cropping on soil nutrients, enzyme activities and microbial community structure of rhizosphere soil in tobacco. Biotechnol. Bull. 41, 312–322. doi: 10.13560/j.cnki.biotech.bull.1985.2024-0645

[B67] Zhao S. Long G. Yang C. Tang L. Zheng Y. (2016). Effects of nitrogen application on nitrogen accumulation and distribution of crops in maize and potato intercropping. J. Yunnan Agric. Univ. (Natural Science) 31, 886–894. doi: 10.16211/j.issn.1004-390X(n)42284679

[B68] Zhao P. Zhou H. Liao X. Zhao L. Zheng Y. Xiong T. . (2025a). The regulation of tobacco growth under preceding crop planting: Insights from soil quality, microbial communities, and metabolic profiling. Front. Plant Sci. 16, 1530324. doi: 10.3389/fpls.2025.1530324 39990714 PMC11842363

[B69] Zhong Y. Liang L. Xu R. Xu H. Sun L. Liao H. (2022). Intercropping tea plantations with soybean and rapeseed enhances nitrogen fixation through shifts in soil microbial communities. Eng. Agric. 9, 344–355. doi: 10.15302/j-fase-2022451

[B70] Zhou Z. Zhang Y. Zhang F. (2022). Abundant and rare bacteria possess different diversity and function in crop monoculture and rotation systems across regional farmland. Soil Biol. Biochem. 171, 108742. doi: 10.1016/j.soilbio.2022.10874238826717

