# Correction: Relay maize after tobacco enhances rapeseed growth and nutrition by reshaping soil microbial communities in an annual triple cropping system

**DOI:** 10.3389/fpls.2026.1935580

**Published:** 2026-10-09

**Authors:** Yuxin Yang, Yongcong Xia, Yuanyuan Li, Wenwu Li, Jiajie Luo, Cunwu Guo, He Zhang, Bingbing Jiang, Shusheng Zhu, Junying Li, Yixiang Liu, Cuiying Wang

**Affiliations:** 1 Baoshan University, College of Resources and Environment, Baoshan, China; 2 Yunnan Agricultural University, Key Laboratory of Agricultural Biodiversity and Pest Control of Ministry of Education/State Key Laboratory of Conservation and Utilization of Yunnan Biological Resources, Kunming, China; 3 Yunnan Academy of Tobacco Agricultural Sciences, Kunming, China; 4 International Associated Laboratory of China and French in Agriculture, Yunnan Agricultural University, Kunming, China; 5 Linxiang District Agricultural Technology Extension Station, Lincang, China

**Keywords:** growth dynamics, plant nutrition, rapeseed, soil microbial, theoretical yield, triple-cropping system

There was a mistake in **Figures 3**, **5** as published. Due to a coefficient conversion issue during data processing, the total potassium (TK) values for rapeseed were incorrectly calculated, and the Pearson correlation coefficients in the correlation matrix were subsequently affected. The corrected **Figures 3**, **5** appear below:

**Figure 3:**
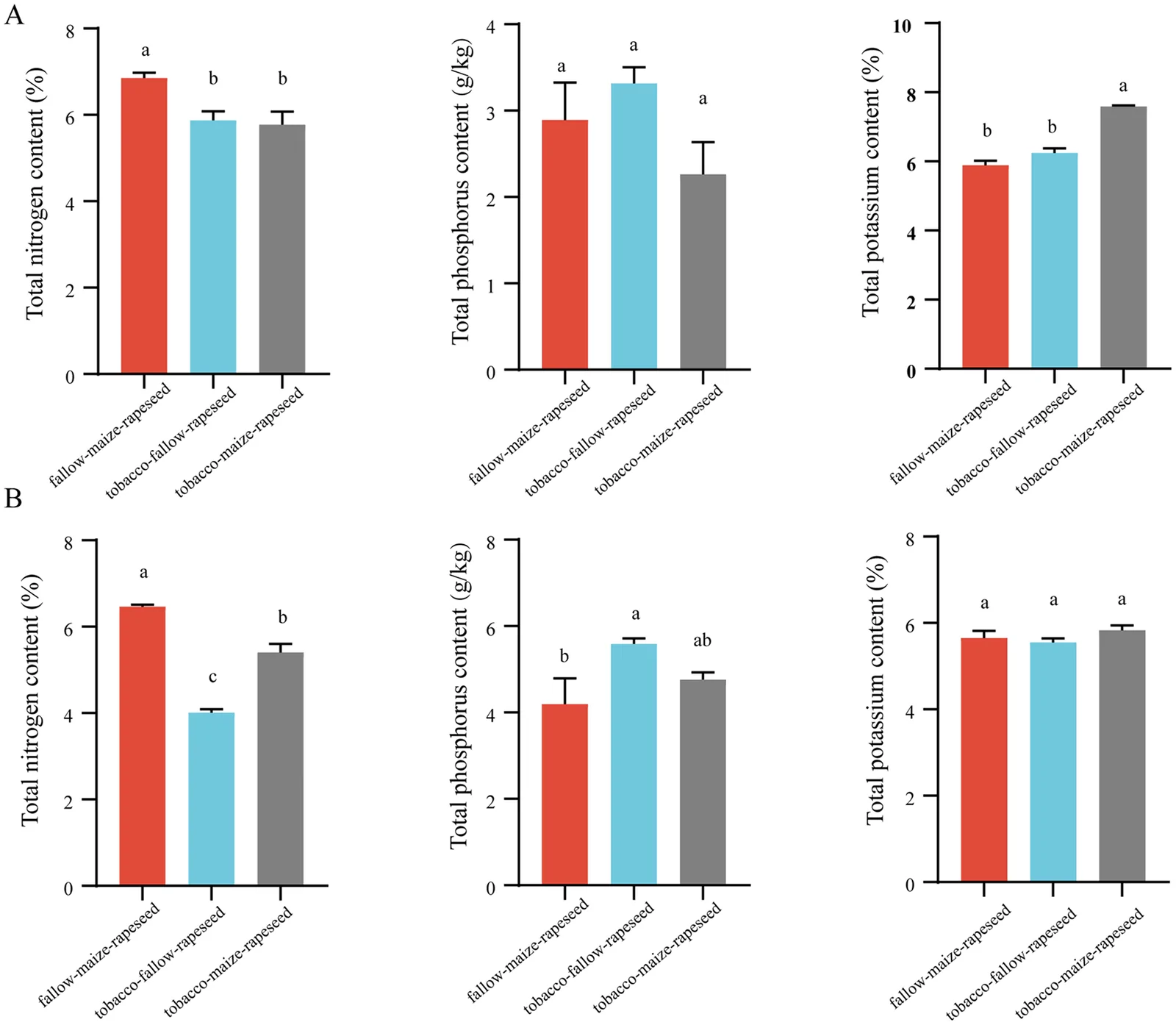
Effects of different cropping systems on nutrient contents in rapeseed plants. **(A)** TN, TP, and TK contents at the seedling stage of rapeseed; **(B)** TN, TP, and TK contents at the flowering stage. Error bars represent standard error (SE) of the mean (n = 3). Different lowercase letters indicate significant differences among treatments within the same growth stage based on one-way ANOVA followed by Duncan's new multiple range test (P < 0.05). F_M_R, fallow–maize–rapeseed; T_F_R, tobacco–rapeseed rotation; T_M_R, tobacco–rapeseed rotation with an additional relay maize crop.

**Figure 5:**
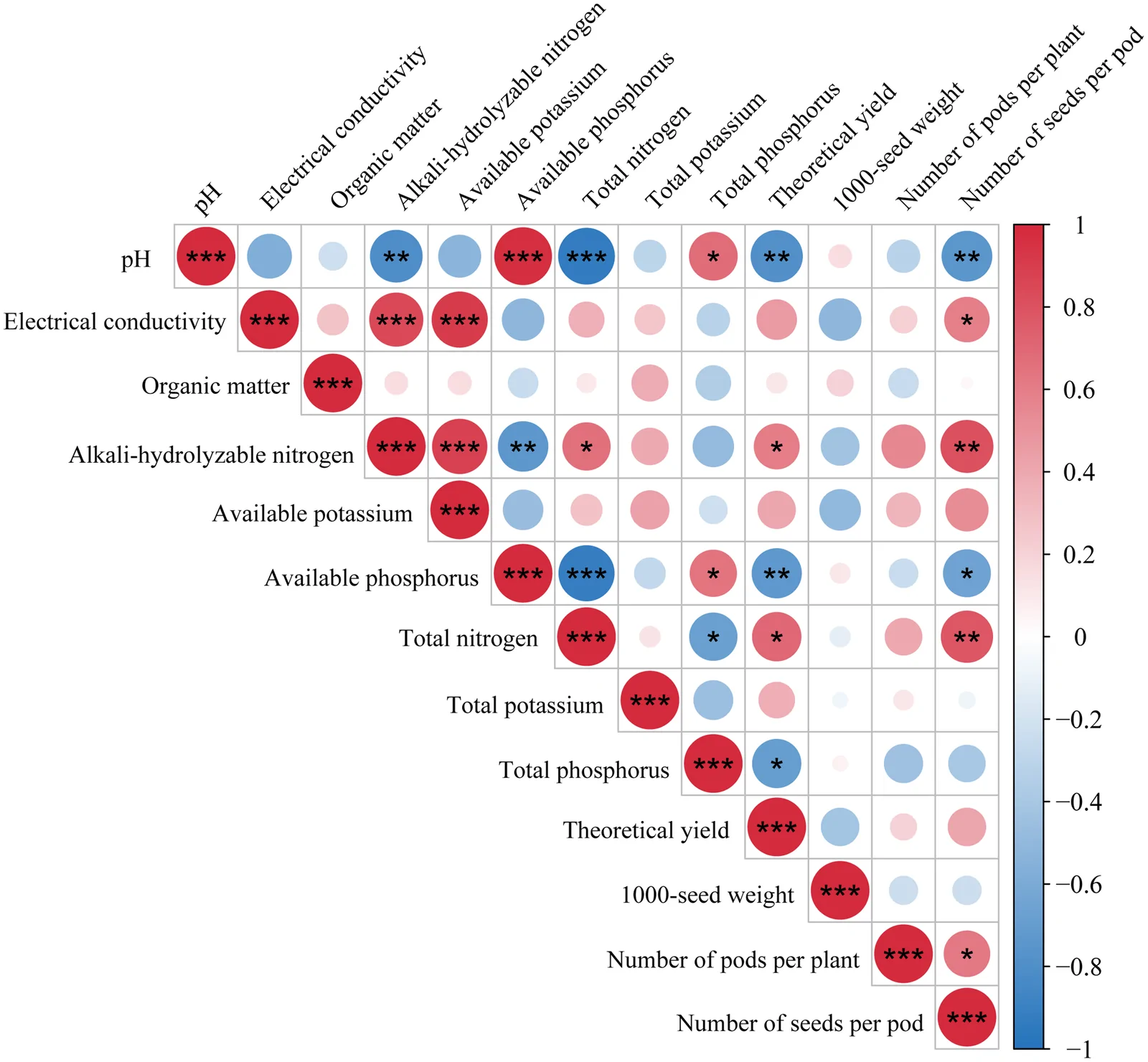
Correlation of soil physicochemical properties and plant nutrients with rapeseed theoretical yield and yield components. Correlation analyses were performed using Pearson’s correlation coefficient. The circle size represents the magnitude of the correlation coefficient; red indicates positive correlation, and blue indicates negative correlation. *, **, and *** denote significance at the levels of P < 0.05, P < 0.01, and P < 0.001, respectively.

In the published article, the captions for **Figures 1, 2** were mismatched with their respective figures. The corrected captions appear below:

“Figure 1. Logistic growth curves of plant height and dry weight of rapeseed under different cropping systems.

Figure 2. Instantaneous growth rate curves of plant height and dry weight of rapeseed under different cropping systems.”

In the captions of **Figures 3**, **4**, and in the footnote of **Table 1**, the *post-hoc* multiple comparison test was erroneously given as “Tukey’s multiple comparison test” instead of “Duncan’s new multiple range test at P < 0.05”. Also, the footnote of **Table 1** has been standardized to “Data are presented as mean ± standard error (n = 3)”. The corrected captions and footnote appear below:

“Figure 3. Effects of different cropping systems on nutrient contents in rapeseed plants. **(A)** TN, TP, and TK contents at the seedling stage of rapeseed; **(B)** TN, TP, and TK contents at the flowering stage. Error bars represent standard error (SE) of the mean (n = 3). Different lowercase letters indicate significant differences among treatments within the same growth stage based on one-way ANOVA followed by Duncan’s new multiple range test (*P* < 0.05). F_M_R, fallow–maize–rapeseed; T_F_R, tobacco–rapeseed rotation; T_M_R, tobacco–rapeseed rotation with an additional relay maize crop.”

“Figure 4. Soil physicochemical properties under different cropping systems before rapeseed planting. Error bars represent standard error (SE) of the mean (n = 3). Different letters indicate significant differences among treatments based on one-way ANOVA followed by Duncan’s new multiple range test (*P* < 0.05). F_M_R, fallow–maize–rapeseed; T_F_R, tobacco–rapeseed rotation; T_M_R, tobacco–rapeseed rotation with an additional relay maize crop.”

“Table 1. Yield components and productivity of rapeseed under different multiple cropping systems. Data in the table as the mean ± standard error (n = 3). Different lowercase letters indicate significant differences among different multiple cropping systems based on one-way ANOVA followed by Duncan’s new multiple range test (*P* < 0.05). T_M_R, tobacco–rapeseed rotation with an additional relay maize crop; T_F_R, tobacco–rapeseed rotation; F_M_R, fallow–maize–rapeseed.”

A correction has been made to section **2 Materials and methods,** *2.4 Determination of rapeseed yield components and theoretical yield*. “ha” should be used instead of “hm^2^”. The corrected text appears below:

“Theoretical yield (kg/ha) = Number of effective plants per hectare × number of pods per plant × number of seeds per pod × 1000-seed weight × 10^-6^ × 0.85”.

A correction has been made to section **3 Results,** *3.1 Effects of different multiple cropping patterns on rapeseed yield components and theoretical yield*, last sentence. “than” should be added before “that”. The corrected text appears below:

“Rapeseed theoretical yield with the addition of a maize season (T_M_R) reached 5388.48 kg/ha, which was significantly higher than that of the conventional T_F_R treatment, with no significant difference compared with the F_M_R treatment”.

A correction has been made to section **3 Results,** *3.4 Effects of different multiple cropping patterns on the nutrition of rapeseed plants*, first paragraph. *“*7.59%” should replace “25.39%”. The corrected text appears below:

“In contrast, total potassium (TK) content varied among treatments, with the highest value observed under the T_M_R treatment (7.59%)”.

The original version of this article has been updated.

