# Supplementary material for: Relay maize after tobacco enhances rapeseed growth and nutrition by reshaping soil microbial communities in an annual triple cropping system
Source: Front Plant Sci. 2026 Jul 2;17:1827060. doi: 10.3389/fpls.2026.1827060 (PMC13373781; doi:10.3389/fpls.2026.1827060)
Supplement: Supplementary Table 1 — Initial physicochemical properties of the experimental soil. [file Table1.docx]

**Supplementary Material**

**Table S1. Initial physicochemical properties of the experimental soil**

| Parameter | pH | organic matter  (g/kg) | Electrical conductivity (μS/cm) | Available nitrogen (mg/kg) | Available phosphorus (mg/kg) | Available potassium (mg/kg) |
| --- | --- | --- | --- | --- | --- | --- |
| Value | 6.95±0.05 | 20.45±0.53 | 102.27±21.51 | 95.18±6.91 | 12.57±0.64 | 167.53±14.92 |

Values represent the initial soil properties measured before the establishment of the experiment. Data in the table as the average number ± standard error (n=3).
