# Supplementary figures and images for: Relay maize after tobacco enhances rapeseed growth and nutrition by reshaping soil microbial communities in an annual triple cropping system
Source: Front Plant Sci. 2026 Jul 2;17:1827060. doi: 10.3389/fpls.2026.1827060 (PMC13373781; doi:10.3389/fpls.2026.1827060)

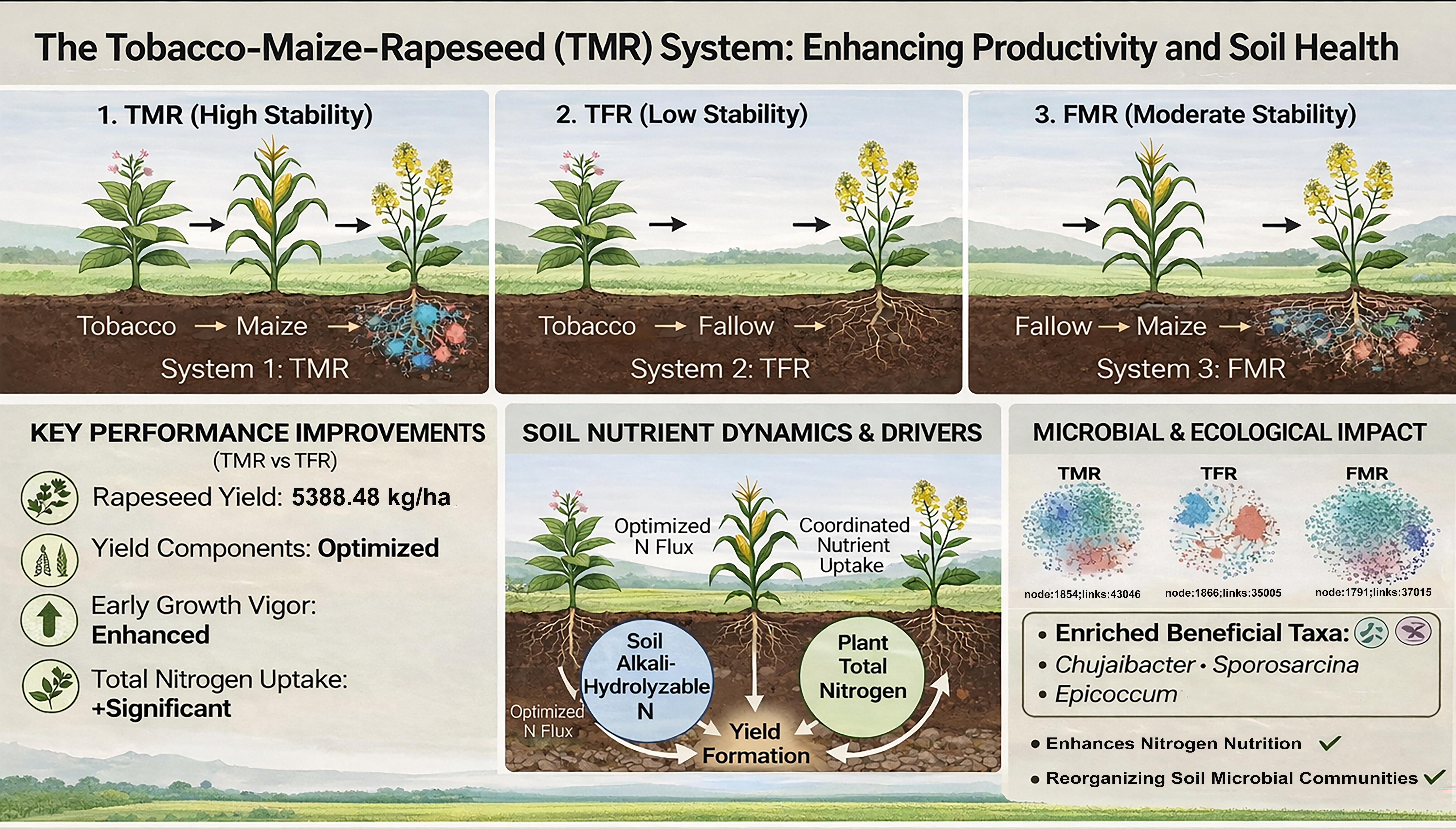

Supplement: Supplementary file 2 [file Image1.jpeg]
